# Metal-polyphenol polymer modified polydopamine for chemo-photothermal therapy

**DOI:** 10.3389/fchem.2023.1124448

**Published:** 2023-01-24

**Authors:** Li Xu, Zhibing Luo, Qing Liu, Chuancui Wang, Fei Zhou, Min Zhou

**Affiliations:** ^1^ Department of Respiratory Medicine, Jinshan District Central Hospital affiliated to Shanghai University of Medicine & Health Sciences, Shanghai, China; ^2^ Department of Pulmonary and Critical Care Medicine, Shanghai East Hospital, Tongji University School of Medicine, Shanghai, China; ^3^ Department of Medical Oncology, Shanghai Pulmonary Hospital, Thoracic Cancer Institute, Tongji University School of Medicine, Shanghai, China

**Keywords:** polydopamine, Fe-Ga, photothermal therapy, Paclitaxel, lung cancer

## Abstract

Chemotherapy combined with photothermal therapy (PTT) is a new way to improve the curative effect of cancer treatment. Here, we developed a multifunctional nanoparticle, namely PTX@mPDA@Fe-GA with the loading of a chemotherapeutic drug paclitaxel (PTX) for targeted and synergistic chemotherapy/photothermal therapy in lung cancer. Fe-gallic acid (Fe-GA) was coated on the surface of mesoporous polydopamine (mPDA) nanoparticles, and then the PTX was placed in the mesopores. The drug release of the loaded PTX exhibited pH- and thermal-dual responsive manner. Both mPDA and Fe-GA have high photothermal conversion ability and play a role in photothermal therapy. In addition, the results revealed that mPDA@Fe-GA had excellent biocompatibility and low hemolysis rate. The PTX-loaded mPDA@Fe-GA not only has excellent killing effect on lung cancer cells (A549) *in vitro*, but also can significantly suppress the growth of A549 subcutaneous tumor in nude mice. In a nutshell, the developed multifunctional nanoparticles integrate photothermal therapy and efficient chemotherapeutic drug delivery, providing new therapeutic ideas in the fight against lung cancer.

## 1 Introduction

The morbidity of lung cancer is increasing year by year, and it almost ranks the first in the morbidity of malignant tumors (Brody 2020). There are many methods for the treatment of malignant tumors, but the main three methods are surgery, radiotherapy and chemotherapy, supplemented by targeted therapy, gene therapy, immunotherapy and biological therapy (Hirsch et al., 2017; Duma et al., 2019; Miller and Hanna 2021). However, various treatment methods have their own limitations, such as large surgical trauma, many complications, long postoperative recovery period, radiotherapy and chemotherapy can effectively kill tumor cells, but also have great toxic side effects on normal tissues and organs of the body, targeted therapy is easy to produce drug resistance, and so on (Waqar and Morgensztern 2017; Osmani et al., 2018; Bogart et al., 2022). Therefore, it is the most urgent to develop highly effective and selective anticancer drugs with few side effects.

Nanomedicine is emerging as a new method that does not produce drug resistance, can prevent tumor metastasis and recurrence, and has low toxicity (Markman et al., 2013; Hussain 2016; Li et al., 2021). It was recently shown that nanomedicine endocytosis has better treatment effect and weak side effects after accumulation in tumor tissues through passive enhanced permeability and retention (EPR) action of tumors (Nehoff et al., 2014). In addition, the new nanomedicine should be capable to release the delivered chemotherapy in a controlled manner (Heleg-Shabtai et al., 2016). At present, researchers have explored many smart responsive nanomedicine, such as nanomedicine that responds to external stimuli (light, magnetic field, ultrasound, etc.,) or internal stimuli (pH, temperature, enzyme, REDOX potential, etc.,) (Mazrad et al., 2018; Qiao et al., 2019; Zhao et al., 2021). Massive researches have indicated that the pH value of tumor tissue is 6.5–6.9, which is lower than 7.2–7.4 of adjacent tissues and normal tissues (Tang et al., 2018; Huo et al., 2019). This difference is caused by the high rate of aerobic glycolysis (Warburg effect) and the production of lactate and protons in tumor tissue (Dominski et al., 2020). In view of the characteristic of low pH value in tumor microenvironment, pH-responsive nanomedicine has a wide application prospect.

Photothermal therapy (PTT) utilizes the photothermal effect of photothermal agents (PTAs) to convert the absorbed light energy into heat to cause thermal burns on tumors (Zhi et al., 2020; Yue and Zhao 2021). PTT has the advantages of simple operation, short treatment time and rapid recovery, which has high research value (Chen et al., 2020). Photothermal reagents mainly include organic photosensitive molecules (indocyanine green, methylene blue, etc.,) and inorganic materials (precious metal nanoparticles (NPs), metal sulfide nanomaterials, carbon nanomaterials, quantum dots, etc.,) (Chang et al., 2019). However, organic photosensitive molecules can be eliminated quickly in the blood and cannot be selectively enriched in tumor regions, and inorganic materials have poor biocompatibility (Sun et al., 2021). Polydopamine (PDA) is a main ingredient of natural biological pigment melanin, and its derived nanomaterials exhibit excellent stability, biodegradability, biocompatibility, and photothermal conversion properties (Zhang P. et al., 2021; Lu et al., 2021). In addition, coordination polymers composed of Fe (III) and gallic acid (GA) have been widely used in drug delivery, where Fe (III) coordinates with the -COOH of GA in an unsaturated mode (Mu et al., 2017). It can be gradually degraded under the effect of a weakly acidic tumor microenvironment (TME) and effectively discharged from the body without causing long-term retention and toxicity problems (Liu Z. et al., 2021). However, the drug loading capacity of Fe-GA coordination polymers mainly relies on the surface adsorption that relies on between drug molecules and Fe-GA, resulting in suboptimal drug loading capacity. Consequently, there is an urgent need to design Fe-GA complexes with larger lumen for drug loaded tumor therapy, while mPDA has sufficient mesoporous space.

**FIGURE 1:**
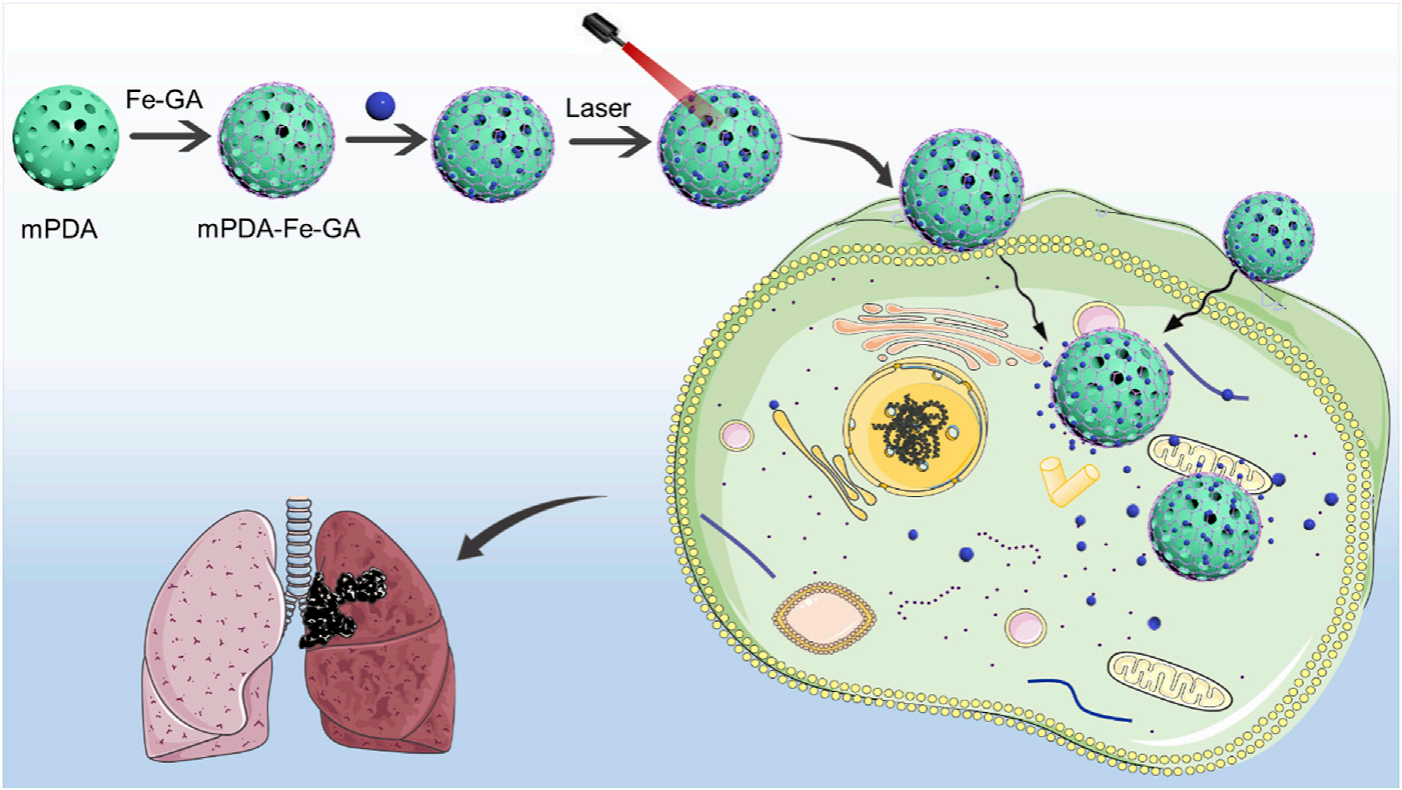
The preparation process and schematic diagram of PTX@mPDA@Fe-GA. PTX, paclitaxel; mPDA, mesoporous polydopamine; Fe-GA, Fe-gallic acid.

In order to use nanomedicine for the efficient treatment of lung cancer, we constructed a functional nanoplatform coated with mPDA and Fe-GA for the combined photothermal chemotherapy treatment of liver cancer. Considering the high porosity and easy functionalization of mPDA nanoparticles, mPDA nanoparticles with mesoporous channels were used as supercarriers, coated with Fe-GA and mesoporous loaded PTX. In summary, PTX@mPDA@Fe-GA nanoparticles have good stability and biocompatibility, can significantly inhibit the growth of lung cancer cells *in vitro* and *in vivo* experiments, and achieve good tumor treatment efficiency. In the process of animal experiments, PTX@mPDA@Fe-GA nanoparticles also show no obvious toxicity. This study finally realized the combined tumor therapy of PTX@mPDA@Fe-GA nanomaterials (Figure 1).

## 2 Experimental section

### 2.1 Materials

F127, FeCl_2_·4H_2_O, and 1,3,5-tetramethylbenzidine (TMB) were purchased from Sigma-Aldrich (St. Louis, MO, United States). All chemicals were of analytic grade and used without further purification. A549 lung cancer cells were purchased from ATCC (Manassas, VA, United States). Ethanol, acetone, triethylamine, and PVP were purchased from Aladdin (Shanghai, China); Paclitaxel was purchased from Macklin (Shanghai, China); DMEM was purchased from Gibco (Grand Island, NY, United States); FITC, DAPI, CCK-8 Kit, and DCFH-DA were purchased from Beyotime Biotechnology (Shanghai, China).

### 2.2 Preparation of mPDA nanoparticles

The mPDA nanoparticles were synthesized by a one-pot synthesis method by previously reported procedures (Guan et al., 2016). Firstly, 0.3 g of dopamine and 0.20 g of F127 were dissolved in 50% ethanol solution and stirred at room temperature. Then, 0.32 mL TMB was added to the mixture. After sonication in a water bath for 2 min, the mixture gradually formed an emulsion solution. Immediately, quickly add 0.75 mL ammonia to the reaction mixture with stirring. After 2 h of reaction at RT, the resultant mPDA nanoparticles were gathered by centrifugation and washed for 3 times with ethanol and water. The template was removed by extraction, and the prepared PDA nanoparticles were sonicated for 0.5 h (3 times) in a mixed solvent of ethanol and acetone (v/v, 2/1). Lastly, the resulting mPDA nanoparticles were re-dispersed into ultrapure water.

### 2.3 Fe-GA coating

To synthesize mPDA@Fe-GA nanoparticles, 100 μL GA aqueous solution (20 mg/mL) and 10 μL triethylamine (TEA) were added into 4 mL mPDA solution (5 mg/mL in ethanol) containing 40 mg PVP and then vigorously stirred at RT for 5 min. Then, 1, 2, 4, or 8 mg FeCl_2_·4H_2_O dissolved in 200 μL water was added to the reaction mixture and the stirring was continued for 0.5 h. Lastly, the resulting mPDA@Fe-GA nanoparticles were gathered and washed by centrifugation with ethanol at 13,000 rpm.

### 2.4 Loading capacity of PTX

For PTX@mPDA@Fe-GA, PTX was added into the mPDA@Fe-GA solution and mixed in the dark for 24 h. To detect the loading capacity of PTX, 1 mL mPDA@Fe-GA (100 μg/mL) was dissolved in DI water and mixed with different weight of PTX (100, 50, 25, 12.5, 6.25 μg), respectively. Mix the mixture with different weight ratios continuously for 24 h. All product solutions were purified at 14,000 rpm for 20 min. After stirring and washing, unconjugated PTX was collected and quantified through the standard curve. The final loading capacity was calculated by the following equation: (weight of total PTX-weight of free PTX)/(weight of total PTX-weight of free PTX + weight of mPDA@Fe-GA).

### 2.5 Characterization

SEM images of mPDA were observed by Hitachi S-4800 high resolution scanning electron microscope (acceleration voltage 1.0 kV; Hitachi; Japan). TEM images were observed by JEOL JEM-2100 F high-resolution (voltage 200 kV; JEOL Ltd., Janpan).

The changes of particle size after coating Fe-GA and loading PTX drug were observed by Dynamic Light Scattering (DLS). Firstly, appropriate sample was diluted 3 times with ultrapure water. Sample was filtered with 0.22 μm microporous filter membrane after mixing. Then, sample was washed and add a test sample of 2/3 device height. Finally, cover was closed, and tested Size and Zeta potential.

### 2.6 mPDA@Fe-GA biocompatibility

The mixture of 3 mL blood and PBS (v/v, 1/2) was centrifuged at 8000 rpm for 10 min. After Adding 3 mL PBS to wash 5 times, red blood cells were dispersed in 10 mL PBS. Red blood cells were added to PBS as negative control, and red blood cells were added to deionized water as positive control. 0.2 mL of red blood cell dispersion was mixed with 0.8 mL of different concentrations of mPDA and mPDA@Fe-GA. After it mixed evenly, the samples were allowed to stand at room temperature for 3 h, and then all samples were centrifuged (12,000 rpm, 5 min). The UV supernatant were observed and measured. Hemolysis rate calculation formula: hemolysis rate = (Abs _sample_-Abs _negative control_)/(Abs _positive control_-Abs _negative control_).

### 2.7 PTX@mPDA @FE-GA drug release rate

Dialysis bags (16 mm, MWCO:3500Da) were added with 1 mg/mL PTX@mPDA@Fe-GA solution, and the dialysis bags were placed in a beaker filled with PBS solution (pH 5.5 or pH 7.4). The temperature in the beaker was maintained at 37°C, and the beaker was continuously stirred in the dark. After 0, 2, 4, 6, 8, 10, 12, 24, 48, 72 h, 1 mL liquid in the beaker was taken out and supplemented with the same volume of PBS, and the concentration of PTX was measured by high performance liquid chromatography (HPLC).

### 2.8 Photothermal properties

The concentration of mPDA@Fe-GA was 0.1 mg/mL, and the infrared laser with power of 808 nm was irradiated by 0.5 and 0.3 w/cm^2^ laser for 5 min. The initial temperature concentration was 23°C (pure water temperature rise was ignored). The power intensity of the irradiation mPDA@Fe-FA laser was changed and the concentration of 0.1 mg/mL was irradiated for the same time. Thermal stability was assessed by irradiating with mPDA@Fe-FA solution in the near infrared (808 nm, 0.5 w/cm^2^), and temperature change curves were recorded during a heating and cooling process for imaging. After laser irradiation, the experiment was cooled to RT and the parallel experiment was repeated.

### 2.9 Cell culture

Human non-small cell lung cancer cell line (A549) was cultured in DMEM supplemented with 10% fetal bovine serum (FBS) and 0.1% penicillin–streptomycin at 37°C cell incubator with 5% CO_2_. A549 cells were digested with 0.25% trypsin every 1-2 days, centrifuged at 13,000 rpm for 5 min to subculture.

### 2.10 Cellular uptake

A549 cells in logarithmic growth phase were spread in 24-well plates and cultured overnight in an incubator at 37°C, 5% CO_2_. After removing the original medium, FITC-labeled mPDA and FITC- labeled mPDA@Fe-GA were added and incubated for 6 h. PBS (FITC-labeled) was control group. Wash 3 times with PBS and add 4% polyformaldehyde at room temperature to fixed 15 min. After washing with PBS, DAPI was added and incubated at 37°C for 10 min. The uptake of material by A549 cells was observed by fluorescence microscopy.

### 2.11 Cytotoxicity

A549 cells were incubated in 96-well plates with 100 μL of cell suspension at 37°C and 5% CO_2_ incubator for 24 h 1 mg/mL mPDA@Fe-GA were added for 24 h, and 10 μL CCK-8 was added to each well and incubated for 4 h. The absorbance value of cells at 450 nm was measured by microplate and the cell survival rate was calculated. Cell viability (%) = [A (dosing)-A (blank)]/[A (0 dosing)-A (blank)] × 100%, where A (dosing) is the absorbance of Wells adding drug solution, A (blank) is the absorbance of Wells without cells, and A (0 dose) is the absorbance of Wells without drug solution.

### 2.12 Detection of intracellular ROS

A549 cells were digested with trypsin and seeded in 24-well plates overnight. Then add 200 μL mPDA NPs, mPDA@Fe-GA and PTX@mPDA@Fe-GA with concentration of 100 μg/mL for 12 h. After irradiating with a laser of 808 nm and power density of 0.5 w/cm^2^ for 5 min, the cells were cultured in an incubator for 4 h. 1 mM DCFH-DA solution was prepared by DMSO, and then diluted 100 times with serum-free medium. Each well was added with 200 μL DCFH-DA solution and cultured for 0.5 h. The DCFH-DA solution was removed and washed 3 times with serum-free medium. Each well was added with 200 μL PBS, and the intracellular green fluorescence intensity was observed under a fluorescence microscope.

### 2.13 Dead and living cell staining test

The 1 × 10^4^ A549 cells were spread on the 24-well plate. The working solution of the probe incubation was diluted: 1 μL AM, 1 μL PI, and 1 mL buffer were added to the 24-well plate (250 μL). Cells incubated at 37°C for 0.5 h, and photographed under a fluorescence microscope.

### 2.14 Lung cancer model establishment

The 5-week-old male nude mice were acquired from Shanghai Laboratory Animal Research Center. The mice were kept and used in accordance with the ethical requirements of Jinshan District Central Hospital affiliated to Shanghai University of Medicine and Health Sciences Animal Ethics Committee for laboratory animals.

A549 cells were grown to 1*10^7^. After washing with PBS buffer, it was suspended in 1 mL PBS buffer. A suspension of A549 cells (5*10^6^ cells/200 μL) was injected subcutaneously into the right axilla of nude mice. Then, 20 mice were randomly divided into five groups (n = 4 of each group). The tumor grew to about 100 mm^3^, and administration was started by tail vein injection. Mice in each group were given normal saline, PTX, mPDA@Fe-GA, PTX@mPDA@Fe-GA, PTX@mPDA@Fe-GA + Laser [808 nm 1 w/cm^2^) for 10 min after 12 h of treatment], and the dose was as follows: (100 μL; 20 mg/mL) for 15 days, 3 days each time, a total of five injections. Tumor size and body weight were measured every 3 days. Finally, in the final experiment, tumor tissues were obtained, weighed and fixed with 4% paraformaldehyde.

### 2.15 H&E staining

Firstly, the heart, liver, spleen, lung and kidney of the mice were placed in a container (30%, 50%, 70%, 80%, 95%, 100% ethanol) for 1 h each. After paraffin embedding, the tissues were cut into 3 μm. Then, the slices were placed in a xylene tank, a 95% alcohol tank, an 85% alcohol tank, and a 75% alcohol tank (5 min per tank). Next, the tissues were stained and sealied. Tissues were stained in hematoxylin solution for 4 min, and washed with running water for 1 min. Tissues were stained in eosin solution for 1 min, and washed with running water. Slices washed with 75% alcohol, 85% alcohol, 95% alcohol, 3 min per cylinder (3 min per cylinder).

### 2.16 Biological safety

Healthy mice were intravenously injected with mPDA @ Fe-GA (100 μL, 20 mg/mL). The main organs (heart, liver, spleen, lung and kidney) of mice were collected on days 1, 7 and 30 after injection (normal mice were used as controls), and the damage of nanomaterials to specific organs was observed by H&E staining (methods refer to the above action).

The blood of each mouse was collected for the detection of aspartate aminotransferase (AST), alanine aminotransferase (ALT), alkaline phosphatase (ALP), blood urea nitrogen (BUN), creatinine (CRE).

### 2.17 Statistical analysis

Results Data were expressed as mean ± standard deviation (X ±SD). The GraphPad Prism 8.0 software (GraphPad Software, San Diego, CA, United States) was performed to the statistical analyses. Methods of Statistical Analysis [*t*-test for comparison between two groups, One-way analysis of variance was used to test the difference between the means of several subgroups of a variable (multiple testing)], *p* < 0.05 was considered statistically significant.

## 3 Results and discussion

### 3.1 Preparation of PTX@mPDA@Fe-GA nanomaterials

**FIGURE 2:**
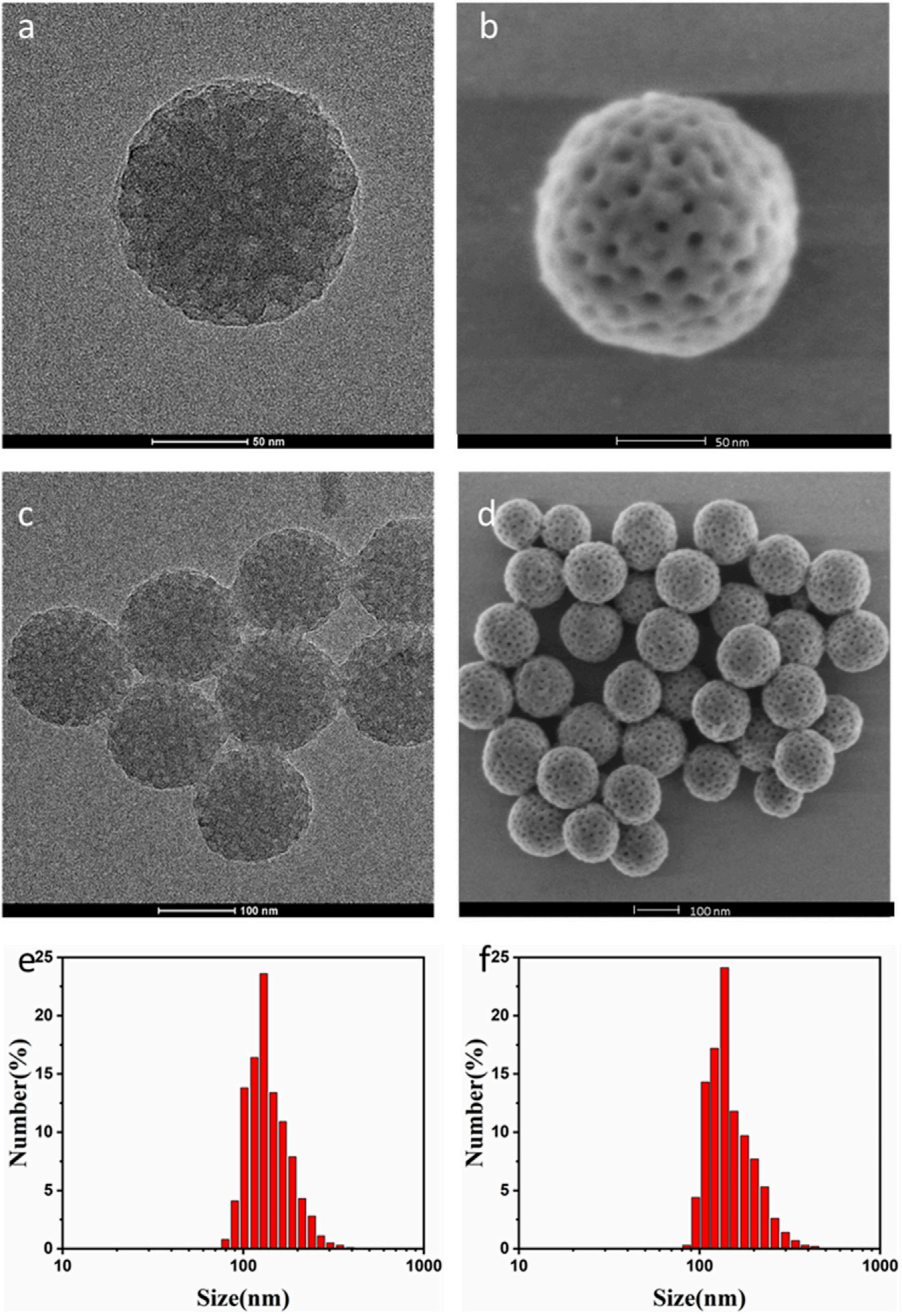
**(A)** TEM image of mPDA single nanoparticles; **(B)** SEM images of mPDA single nanoparticles; **(C)** TEM image of mPDA nanoparticles; **(D)** SEM plot of mPDA nanoparticles; **(E)** The particle size of the mPDA nanoparticles by DLS; **(F)** The particle size of the PTX@mPDA@Fe-GA nanoparticles by DLS. TEM, transmission electron microscopy; mPDA, mesoporous polydopamine; SEM, scanning electron microscopy; DLS, Dynamic Light Scattering.

The synthesis of PTX@mPDA@Fe-GA nanomaterials is shown in Figure 1. Firstly, according to previous reports, mPDA nanoparticles were compounded by one pot method (Guan et al., 2016). mPDA nanoparticles were formed in an aqueous solution containing triblock copolymer Pluronic F-127 and 1, 3, 5-trimethylbenzene (TMB) as organic templates. PDA nanoparticles were self-polymerized by dopamine *via* ammonia catalysis, and assembled on Pluronic f-127 stabilized TMB drops through π—π stacking interaction. Remove the organic template and finally obtain mPDA nanoparticles. TEM (Figures 2A, C) and SEM images (Figures 2B, D) authenticated that the synthesized mPDA nanoparticles have clear spherical morphology and mesoporous structure. Their average diameter and polydispersity were 125 nm (Figure 2E). mPDA nanoparticles have the characteristics of mesoporous structure, large surface area and nanoscale spherical shape, which shows great potential as a multi-functional platform for drug delivery, diagnosis and treatment. It can be clearly observed from Figure 2F and that the particle size of nanoparticles became larger after loading PTX drugs and coating Fe-GA, and the Zeta charge changed to confirm that Fe-GA coating and drug were successfully loaded.

### 3.2 Photothermal effect of mPDA@Fe-GA

**FIGURE 3:**
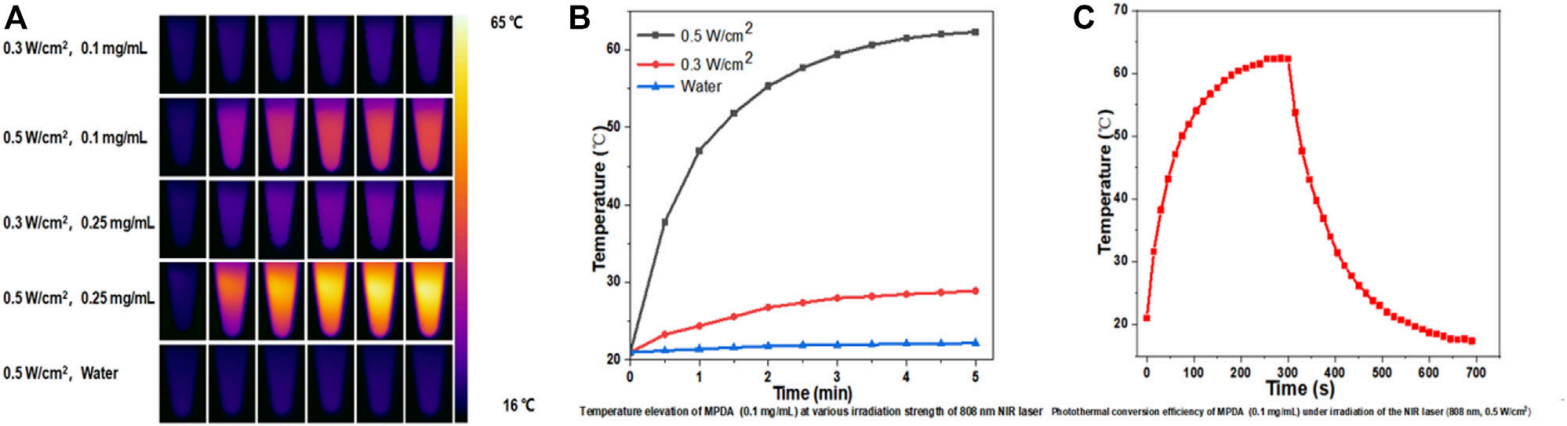
**(A)** The 808-nm laser at 0.3 and 0.5 w/cm^2^ was used to irradiate mPDA@Fe-GA for 5 min and thermal imaging was performed on its aqueous solution; **(B)** Temperature change of 0.1 mg/mL mPDA@Fe-GA under 808 nm laser irradiation with power density of 0.5 and 0.3 W/cm^2^; **(C)** Temperature curve of mPDA@Fe-GA aqueous solution (0.1 mg/mL) during one cycle of near-infrared laser irradiation (808 nm, 0.5 w/cm^2^).

Nanoparticles with high NIR absorption and photothermal conversion efficiency are ideal for PTT. mPDA@Fe-GA nanoparticles exhibit excellent performance in photothermal conversion. Figure 3A showed the infrared thermography of mPDA@Fe-GA aqueous solution continuously rayed by 808 nm laser, the temperature of nanoparticles increases with the concentration and laser irradiation time. After laser irradiation for 5 min, the temperature of 0.1 mg/mL mPDA@Fe-GA aqueous solution was rose from 23°C to 63°C. Under the same laser irradiation condition, the temperature rise of pure water was negligible. In addition, Figure 3B showed that temperature increments of 62.3°C (0.5 W/cm^2^) and 28.9°C (0.3 W/cm^2^) can be adjusted at a concentration of mPDA@Fe-GA of 0.1 mg/mL by simply changing the laser power intensity. These results provided strong evidence that mPDA@Fe-GA effectively absorbs 808 nm laser light and converts it into thermal energy. We next evaluated the photothermal stability by recording the temperature curves of the mPDA@Fe-GA solution during one heating and cooling process under 808 nm laser irradiation. It can be observed from Figure 3C, during the 0–700 s test period, the 300 s reached the highest transformation temperature of 62.4°C and the 700 s cooled, indicating that the significant temperature elevation indicates that mPDA@Fe-GA nanoparticles have excellent photothermal properties and are capable of being used for photothermal therapy of tumors.

### 3.3 Biocompatibility, drug loading and release

**FIGURE 4:**
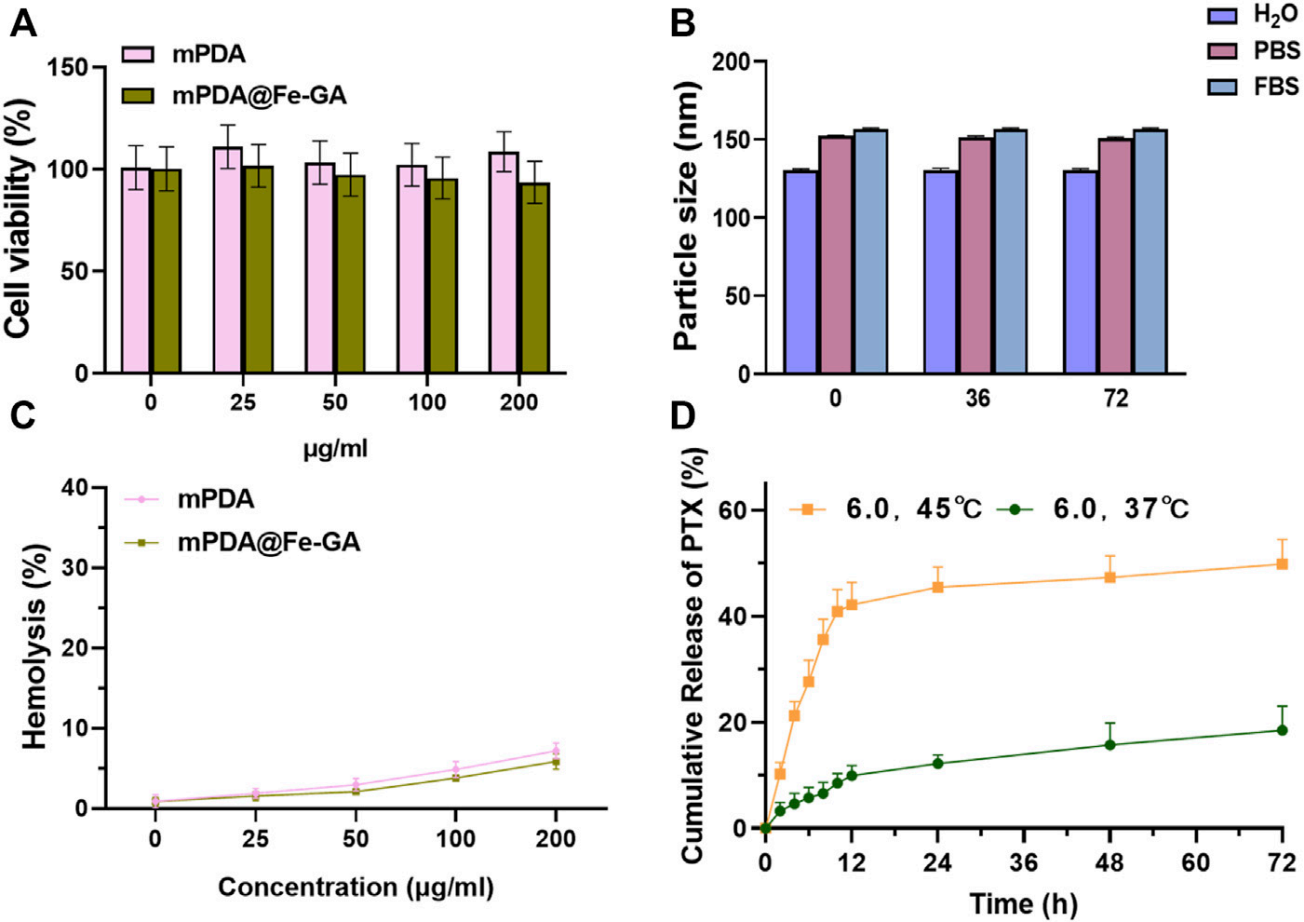
**(A)** CCK-8 was used to detect the toxicity of different concentrations of mPDA and mPDA@Fe-GA on A549 cells; **(B)** Stability of mPDA@Fe-GA in different media detected by DLS; **(C)** Hemolysis detection of mPDA and mPDA@Fe-GA; **(D)** Release efficiency of mPDA@Fe-GA for PTX drugs. CCK-8, Cell Counting Kit-8; mPDA, mesoporous polydopamine; DLS, Dynamic Light Scattering.

The responsive drug delivery and photothermal effect of PTX@mPDA@Fe-GA can be leveraged to kill cancer cells effectively. Thus, cell experiments were performed using human A549 lung cancer cells. Firstly, the cytotoxicity of drug-free mPDA and mPDA@Fe-GA was evaluated *via* the standard CCK-8 assay. A549 cells were incubated with mPDA and mPDA@Fe-GA dispersions at varied concentrations (1, 25, 50, 100, and 200 μg/mL). After 24 h incubation, the relative cell viability was all higher than 85% (Figure 4A), suggesting the excellent biocompatibility of mPDA@Fe-GA. This is consistent with previous studies confirming that mPDA and Fe-GA are non-toxic and have good biocompatibility (Liu C. et al., 2021; Zhu et al., 2021). To verify the stability of mPDA@Fe-GA nanoparticles under physiological conditions, the nanoparticles were placed in water (H_2_O), phosphate buffer (PBS) and culture medium for 0, 36, and 72 h, and then the size of nanoparticles was determined *via* DLS. The results suggested that the average size of mPDA@Fe-GA nanoparticles retained about 135 nm, however the incubation time of 72 h indicated the strong stability of these nanoparticles (Figure 4B). Additionally, the hemocompatibility of mPDA@Fe-GA was preliminarily assessed by the hemolytic assay. Red blood cells were exposed to mPDA@Fe-GA and the absorbance at 540 nm of the collected supernatants was measured to determine the hemolytic percentage. As presented in Figure 4C, the supernatants of all samples were completely transparent, which corroborated that the structural integrity of red blood cells maintained well regardless of the concentration of mPDA@Fe-GA. Moreover, the quantitative result of hemolytic percentage showed that no hemolytic effects occurred even at a high concentration of 200 μg/mL (lower than 5%) (Figure 4C). Therefore, the results demonstrated the good biocompatibility of mPDA@Fe-GA.

The mPDA@Fe-GA has an ideal mesopore structure and serves as a favourable drug delivery vehicle for PTX (Zhang et al., 2019). Then, PTX loading ability of mPDA@Fe-GA nanoparticles were further investigated. mPDA@Fe-GA was dispersed in an ethanol solution, PTX was used as a drug molecule, and unloaded drug was removed by centrifugation and washing. The absorbance of PTX in solution before and after loading was measured by UV-vis, and the drug loading efficiency was about 32.1%. Next, the release ability of mPDA@Fe-GA to PTX was tested through the dialysis bag, and Figure 4D showed the release curves of PTX when PTX@mPDA@Fe-GA was heated to 37°C and 45°C. It should be noted that the total release of PTX was approximately 18.5% at 37°C. In contrast, at 45°C, the release rate of PTX rose sharply, and the release reached 21.2% after 4 h. The early burst release was mainly attributed to the melting of Fe-GA caused by the temperature rise, and the total release of PTX exceeded 49.86% after 72 h. The above experimental results demonstrate that the nanosystem can regulate the release of PTX by controlling the melting of Fe-GA at temperature, which can achieve precise drug release.

### 3.4 *In vitro* therapeutic efficacy of PTX@mPDA@Fe-GA

**FIGURE 5:**
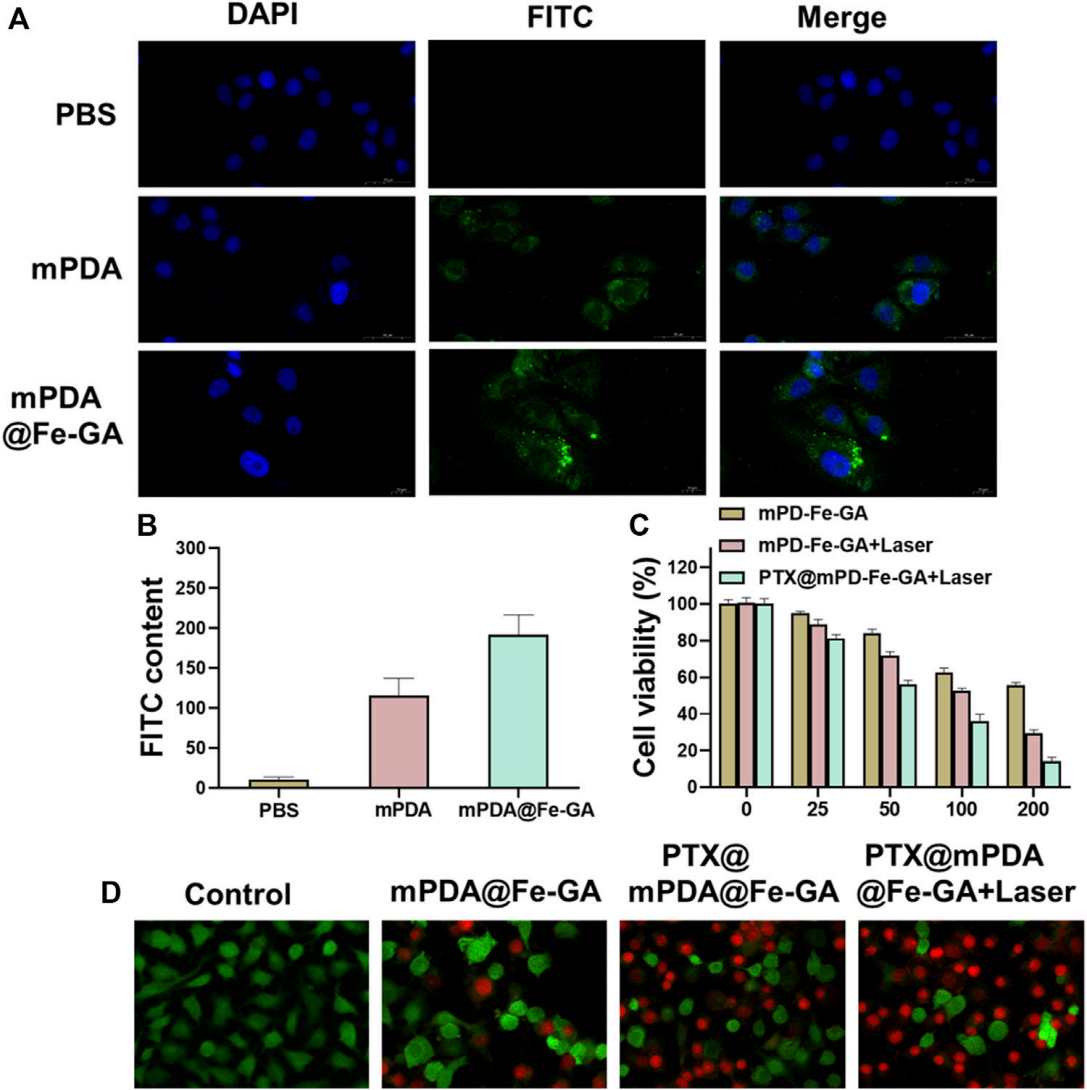
**(A)** CLAM was used to observe the uptake of mPDA@Fe-GA in A549 cells; **(B)** Semi-quantitative statistics of green fluorescence in (A); **(C)** CCK-8 was used to detect cell viability; **(D)** Dead or alive staining of A549 cells.

Nanoparticles should be designed to enter cells rapidly and efficiently to realize a better therapeutic effect. A549 cells were treated with 100 μg/mL mPDA or mPDA@Fe-GA (with FITC probe) for 24 h, respectively, and the uptake of nanomaterials by A549 cells was observed by laser scanning confocal microscopy (LSCM). The results demonstrated that the amount of mPDA@Fe-GA into A549 cells was much higher than that of mPDA (Figure 5A). Meanwhile, Figure 5B confirmed that mPDA@Fe-GA could be efficiently uptaken by A549 tumor cells by fluorescence semi-quantification. To evaluate the anti-lung cancer activity of mPDA@Fe-GA and PTX@mPDA@Fe-GA, CCK-8 assay was performed on lung cancer cell line A549. Different concentrations (0, 25, 50, 100, 200 μg/mL) of nanoparticles were co-cultured with A549 cells for 12 h and then treated with laser irradiation. As shown in Figure 5C, without light treatment, mPDA@Fe-GA and PTX@mPDA@Fe-GA showed no significant difference, and the cell viability gradually reduced with the rise of nanoparticle concentration. Under the laser irradiation, the viability of A549 cells in the PTX@mPDA@Fe-GA group was significantly reduced, and only 35.99% of the cells in the 100 μg/mL group survived, which was significantly lower than that of PTX@mPDA@Fe-GA without laser irradiation at the same concentration, indicating that photothermal therapy can effectively kill cancer cells. When the concentration reached 200 μg/mL and the cells were treated with light, more than 80% of the cells were destroyed, further proving that the chemotherapy-photothermal synergy therapy can effectively inhibit the growth of lung cancer cells.

On the side, the cytotoxicity of the different treatment groups was further observed by dead and live staining. First, A549 cells in 24-well plates were incubated with 100 μg/mL nanoparticles for 12 h. Half of the cells were exposed to an 808 nm laser at 0.5 w/cm^2^ for 5 min, and then stained with Calcem-AM and PI to distinguish live cells from dead cells. As shown in Figure 5D, cells in the blank control group were treated with medium only, and a large number of green fluorescence signals were observed after staining, indicating that all cells remained viable. The amounts of mPDA@Fe-GA and PTX@mPDA@Fe-GA without laser irradiation were slightly decreased compared with the blank control group, which is due to the good biocompatibility of mPDA@Fe-GA without irradiation and the fact that PTX has not been released from mPDA@Fe-GA at room temperature. Using near-infrared laser irradiation of PTX@mPDA@Fe-GA, due to the excellent photothermal properties of the nanoparticles, the number of dead cells was significantly rose by the combined effect of photothermal therapy and chemotherapy under the laser irradiation condition of PTX@mPDA@Fe-GA, which has a better inhibitory effect on A549 cells. It indicated that the released PTX is able to increase the effect of chemotherapy. Laser irradiation can effectively control the release of drugs and decrease the damage to normal cells and tissues. These results clearly demonstrated that PTX@mPDA@Fe-GA has a significant combined therapeutic effect *in vitro*.

### 3.5 ROS detection

**FIGURE 6:**
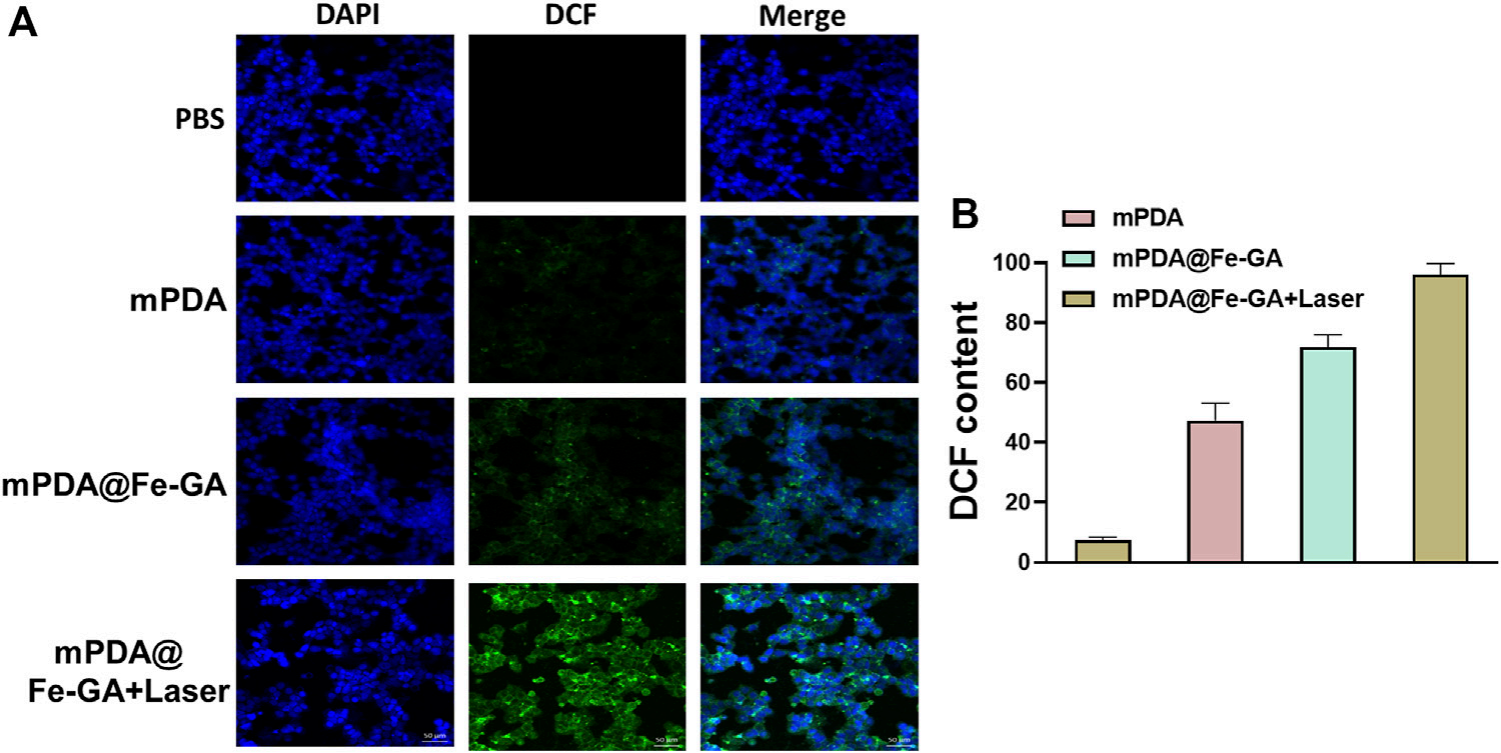
**(A)** DCFH-DA kit detection of ROS in A549 cells after mPDA@Fe-GA and radiotherapy; **(B)** shows the fluorescence semi-quantitative statistics of (A).

Low levels of ROS play an important role in cell proliferation, differentiation, apoptosis, cell signaling, and homeostasis (Wang and Yi 2008; Zhang C. et al., 2021). However, excessive ROS in cells can cause severe damage to DNA, proteins and lipids and is considered to be a signal regulating apoptosis (Srinivas et al., 2019). A549 cells were cultured with PBS, mPDA and mPDA@Fe-GA overnight and then irradiated with laser. The intracellular ROS level was tested by DCFH-DA staining of A549 cells. It was shown in Figures 6A, B, cells in the blank control group did not show green fluorescence, and only weak fluorescence was observed in the mPDA alone group. The mPDA@Fe-GA group showed a significant rise in fluorescence intensity because of the ability of Fe to initiate the Fenton reaction to produce hydroxyl radicals. Under laser irradiation, the maximum fluorescence intensity was reached in the mPDA@Fe-GA group, indicating the greatest ROS production.

### 3.6 Synergistic therapy of AMD *in vivo*

**FIGURE 7:**
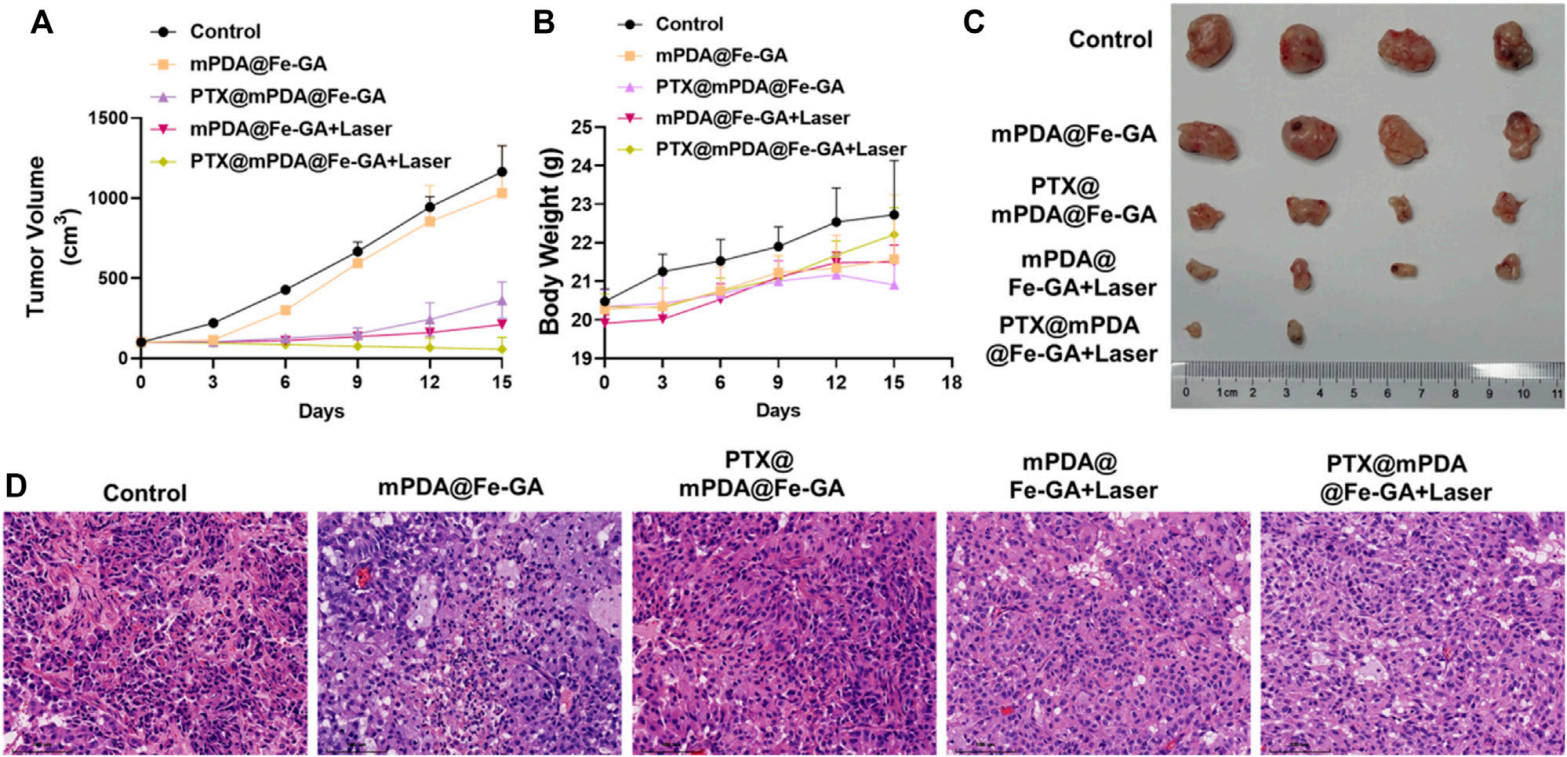
**(A)** Statistical plot of tumor volume; **(B)** Body weight chart of treated mice; **(C)** tumor imaging; **(D)** HE staining. Scale bars = 100 μm for HE staining. HE, Hematoxylin-eosin staining.

Inspired by the anticancer effect of the PTX@mPDA@Fe-GA nanoparticle *in vitro*, we evaluated its *in vivo* therapeutic effect in a A549-bearing nude mouse model. The nanoparticles were injected by tail vein, and 808 nm laser was given at 12 h post-injection for 10 min. As shown in Figures 7A–C, the mice in the PBS or mPDA@Fe-GA groups showed faster tumor growth, indicating that mPDA@Fe-GA did not have much effect on tumor growth. In contrast, the PTX@mPDA@Fe-GA group showed an inhibitory effect on tumor growth, but still could not completely inhibit tumor growth. After mPDA@Fe-GA + Laser treatment, the tumor was further inhibited. The tumors in the PTX@mPDA@Fe-GA + Laser group were completely suppressed, and even two tumors disappeared without recurrence. These results clearly suggest that the prepared PTX@mPDA@Fe-GA nanomaterials can effectively inhibit the tumor growth in mice and can be used as an effective dual-mode photothermal therapy and chemotherapy therapeutic drug. In addition, after 15 days of treatment, we performed H&E staining to evaluate the tumor inhibition of the different treatments. It was shown (Figure 7D) that under the treatment condition of 808 nm NIR irradiation, the tumors treated with PTX@mPDA@Fe-GA nanoparticles had obvious characteristics of apoptosis, vacuolization, nuclear shrinkage and cell number reduction. These results suggested that PTX@mPDA@Fe-GA-mediated synergistic therapy of chemotherapy and PTT could efficiently inhibit the growth of lung cancer.

### 3.7 *In vivo* biosafety evaluation of mPDA@Fe-GA

**FIGURE 8:**
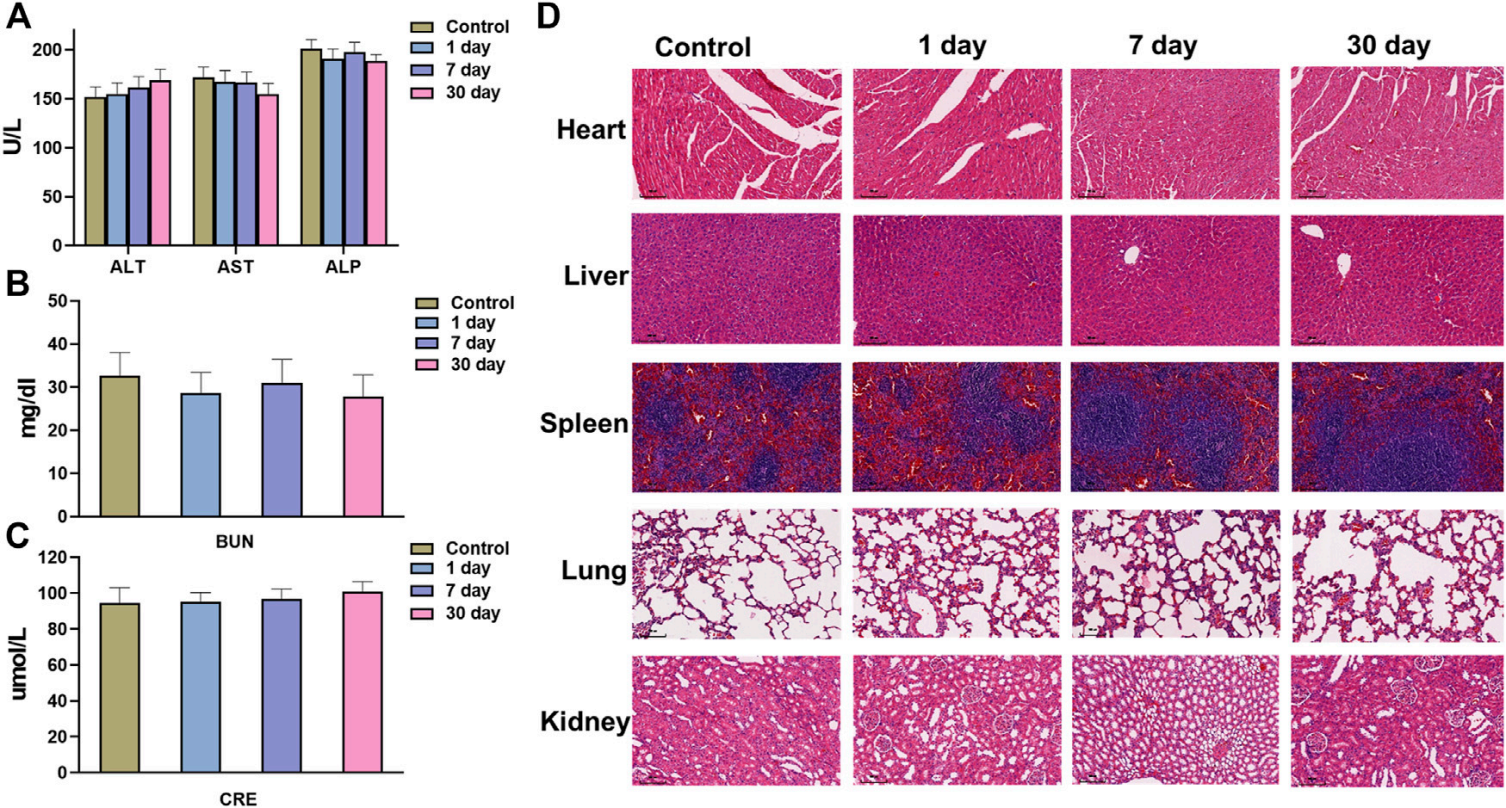
**(A)** AST, ALT, ALP, **(B)** BUN, and **(C)** CRE serum levels in the mice at control, 1, 7 days or 28 days. **(D)** HE staining images of major organs (Heart, Spleen, Lung, Kidney, and Liver) at control, 1, 7 days or 28 days. Scale bars = 100 μm for HE staining. AST, aspartate aminotransferase, ALT, alanine aminotransferase, ALP, alkaline phosphatase, BUN, blood urea nitrogen, CRE, creatinine.

In addition, the potential *in vivo* toxicity of the mPDA@Fe-GA nanoparticles were investigated in terms of blood chemistry and histological evaluation. The dispersion of mPDA@Fe-GA nanoparticles was intravenously injected into healthy Balb/c mice at a dose of 20 mg/kg. Then, the blood of the experimental mice was collected from the eyeballs after 1 day injection. The samples were sent for serum biochemistry examination. As shown in Figures 8A–C, the serum parameters of mPDA@Fe-GA treated mice exhibited no abnormal values as compared with the control group, involving the liver function indexes of alanine aminotransferase (ALT), aspartate aminotransferase (AST) and alkaline phosphatase (ALP), the kidney function markers of blood urea nitrogen (BUN) and creatinine (CRE). Moreover, the major organs (heart, liver, spleen, lung, and kidney) of the treated mice were excreted, sliced, and stained with hematoxylin and eosin (H&E) for histological evaluation after 30 days of intravenous injection mPDA@Fe-GA. The results also suggested that no perceptible tissue damage or inflammatory lesion was observed in all tissue samples, which is similar with the control group (Figure 8D). Thus, the above results demonstrated that the developed mPDA@Fe-GA has negligible toxicity *in vivo*, permitting its safe applications for therapy.

## 4 Conclusion

In order to surmount the difficulties of traditional chemotherapy and enhance the therapeutic effect of lung cancer. In this study, functional inorganic nanocomposites, mesoporous polydopamine and Fe-GA coated nanoparticles (mPDA@Fe-GA) were designed and prepared. When the nanoparticles entered the tumor cells, the tumor site of mice was irradiated with near infrared (mPDA@Fe-GA), which could absorb the incident near infrared light and convert it into heat, and thus realize photothermal therapy. The mesoporous structure of mPDA can carry PTX drugs, which can also rupture under thermal conditions, and sustained release of chemotherapy drugs at the tumor site. The outer Fe ion is conducive to the Fenton reaction, which converts more hydrogen peroxide to hydroxyl radicals. This design realizes the combined treatment of photothermal therapy, chemotherapy and chemical kinetics. In the next, we will combine the previous research to optimize the nanoparticles, giving it excellent tumor-affinity and the therapeutic function to realize targeted diagnosis and therapy.

## Data Availability

The raw data supporting the conclusion of this article will be made available by the authors, without undue reservation.
